# Impaired *In Vivo* Gamma Oscillations in the Medial Entorhinal Cortex of Knock-in Alzheimer Model

**DOI:** 10.3389/fnsys.2017.00048

**Published:** 2017-06-30

**Authors:** Tomoaki Nakazono, Travis N. Lam, Ayushi Y. Patel, Masashi Kitazawa, Takashi Saito, Takaomi C. Saido, Kei M. Igarashi

**Affiliations:** ^1^Department of Anatomy and Neurobiology, University of CaliforniaIrvine, Irvine, CA, United States; ^2^Center for the Neurobiology of Learning and Memory, University of CaliforniaIrvine, Irvine, CA, United States; ^3^Department of Medicine, University of CaliforniaIrvine, Irvine, CA, United States; ^4^Laboratory for Proteolytic Neuroscience, RIKEN Brain Science InstituteWako, Japan; ^5^Japan Science and Technology AgencyTokyo, Japan

**Keywords:** medial entorhinal cortex, gamma oscillations, Alzheimer’s disease (AD), *in vivo* electrophysiology, amyloid precursor protein (APP)

## Abstract

The entorhinal cortex (EC) has bidirectional connections with the hippocampus and plays a critical role in memory formation and retrieval. EC is one of the most vulnerable regions in the brain in early stages of Alzheimer’s disease (AD), a neurodegenerative disease with progressive memory impairments. Accumulating evidence from healthy behaving animals indicates gamma oscillations (30–100 Hz) as critical for mediating interactions in the circuit between EC and hippocampus. However, it is still unclear whether gamma oscillations have causal relationship with memory impairment in AD. Here we provide the first evidence that *in vivo* gamma oscillations in the EC are impaired in an AD mouse model. Cross-frequency coupling of gamma (30–100 Hz) oscillations to theta oscillations was reduced in the medial EC of anesthetized amyloid precursor protein knock-in (APP-KI) mice. Phase locking of spiking activity of layer II/III pyramidal cells to the gamma oscillations was significantly impaired. These data indicate that the neural circuit activities organized by gamma oscillations were disrupted in the medial EC of AD mouse model, and point to gamma oscillations as one of possible mechanisms for cognitive dysfunction in AD patients.

## Introduction

The entorhinal cortex (EC) plays a critical role in the formation of declarative memory (Van Hoesen et al., 1991; Gomez-Isla et al., 1996). The EC interfaces the hippocampus with a number of cortical regions, and most of the cortical input to the hippocampus comes from the EC (Cajal, 1911; Witter and Amaral, 2004). The medial part of EC (medial EC, MEC) is critical in spatial memory (Steffenach et al., 2005), and contains spatially modulated cells including grid cells, head direction cells and border cells (Moser et al., 2014). Information encoded in MEC is sent to hippocampal CA1 via temporoammonic pathway, and to CA3/dentate gyrus via performant pathway, providing spatial information to these regions (Witter et al., 2000).

Accumulating evidence suggests EC as one of the focal sites of disease progression in Alzheimer’s disease (AD), a neurodegenerative disease in which accumulation of amyloid-β (Aβ) and tau leads to synaptic, network and cognitive dysfunctions; In AD patients, the EC shows severe atrophy, with neuronal loss and dense Aβ accumulation (Hyman et al., 1984; Van Hoesen et al., 1991; Gomez-Isla et al., 1996). Functional magnetic resonance imaging studies in both rodents and human suggest neural activity is lost in EC during the early stages of AD (Khan et al., 2014). In addition, a recent study reported reduced MEC grid cell activity in an animal model of AD (Fu et al., 2017). However, it is still largely unclear what types of *in vivo* neuronal activities in the EC are changed by AD.

Gamma oscillations are 30–100 Hz oscillatory activity observed in local field potential (LFP) recordings and derive from transmembrane current of a population of neurons that are periodically synchronized (Buzsáki et al., 2012; Einevoll et al., 2013). In healthy rodents and humans, hippocampus and EC exhibit prominent gamma oscillations that emerge at specific phases of theta (4–12 Hz) oscillations (called theta-gamma cross-frequency coupling; Buzsáki et al., 1983; Soltesz and Deschênes, 1993; Bragin et al., 1995; Chrobak and Buzsáki, 1998; Mormann et al., 2005; Canolty et al., 2006; Colgin et al., 2009). Because oscillatory membrane current of neurons also contributes to spike generation, spike timing normally coincides with specific phases of gamma oscillations (called gamma phase locking of spikes). Computational models suggest that cross-frequency coupling offers an effective mechanism for inter-regional information transfer, because slower theta oscillations can spread to wider areas and synchronize local gamma generators in individual regions (Lisman and Idiart, 1995; Lisman, 2005). Indeed, strength of theta-gamma cross-frequency coupling in hippocampus correlated with the work load of a working memory task in human (Axmacher et al., 2010). In rats, the degree of theta-gamma coupling in the hippocampus correlated with task demands (Tort et al., 2009). The strength of theta-gamma cross-frequency coupling in the EC and CA1 developed during olfactory association learning in rats (Igarashi et al., 2014). These experimental studies indicate that theta-gamma cross-frequency coupling in the entorhinal-hippocampal circuit plays a critical role in memory.

Although an impairment of *in vivo* gamma oscillations in hippocampus and parietal cortex was shown in studies using AD mouse models (Verret et al., 2012; Gillespie et al., 2016; Iaccarino et al., 2016), it is unknown if i*n vivo* gamma oscillations and their temporal structure to theta oscillations are affected in the EC. We therefore evaluated LFP activity and organization of gamma oscillations in MEC using a novel knock-in (KI) mouse model of AD (Saito et al., 2014). This model has a humanized amyloid precursor protein (APP) gene with a triple mutation that causes familial AD in the endogenous APP locus. APP-KI mice are expected to mimic human AD pathology more precisely because: (i) data variation caused by drift of transgene copy number will be eliminated, thus data will be more reliable; and (ii) APP-KI mice have less artificial effects caused by the overexpression of non-Aβ APP fragments which occur in transgenic models. Homozygous APP-KI mice show Aβ deposition in most brain areas at 4-months old, and impairment of performance in a Y-maze memory task at 6-months old (Saito et al., 2014). In this study, we asked if gamma oscillations were affected in 5-month old APP-KI mice.

## Materials and Methods

### Subjects

Data were obtained from five homozygous APP-KI in mice maintained on a C57BL/6 background (Saito et al., 2014; three males and two females) and five non-litter C57BL/6 wild-type (WT) mice (three males and two females). We noted no difference in our data between sexes. Sample size was determined as the minimal number of animals that would provide statistical power to detect a group difference. All animals were 5 months old (25–35 g) at the time of the experiment. Animals were housed in a reversed 12 h light—dark cycle, and all testing occurred during the dark phase. All procedures were conducted in accordance with the guidelines of the National Institutes of Health and approved by the Institutional Animal Care and Use Committee at the University of California, Irvine.

### Electrode Placement and Surgery

Tetrodes were constructed from four twisted 17 μm polyimide-coated platinum-iridium (90%–10%) wires (California Fine Wire). The electrode tips were plated with gold to reduce electrode impedances to between 150 kΩ and 300 kΩ at 1 kHz. During implantation surgery and recording, the animals were anesthetized with urethane (0.6 g/kg mice). A local anesthetic (Xylocaine) was applied on the skin before making the incision. Microdrives containing a bundle of four tetrodes were fixed to a manipulator and inserted into MEC (AP 4.0 mm, ML 3.1 mm from bregma, tetrode tips pointing in the posterior direction at an angle of 20° in the sagittal plane). A screw inserted above the cerebellum was used as a reference and ground electrode during recordings. Tetrodes were lowered in steps of 40 μm. If the estimated tetrode position reached MEC and well separated neurons appeared, we recorded neuronal signals for at least 20 min. After the recording, we further lowered the tetrodes by at least 80 μm to prevent the recording from the same neurons. If well separated neurons appeared, we recorded the signals again. This process was repeated until the estimated tetrode position reached to the layer I of MEC and cellular activity disappeared. All tetrode locations were verified histologically, and the positions at the time of recording were calculated from the final position of the electrodes. Only animals with tetrodes in the dorsal part of MEC were used in this study.

### Data Collection

Spike activity was recorded against tetrodes with no spike activity. LFPs were always recorded single-ended against a ground in the skull above the cerebellum. The tetrodes were connected to a multichannel, impedance matching, unity gain headstage. The output of the headstage was conducted to a data acquisition system (Neuralynx). Unit activity was amplified by a factor of 3000–5000 and band-pass filtered from 600 Hz to 6000 Hz. Spike waveforms above a threshold set by the experimenter (~55 μV) were time-stamped and digitized at 32 kHz for 1 ms. LFP signals, 1 per tetrode, were recorded in the 0–475-Hz frequency band at a sampling rate of 2000 Hz. Notch filters were not applied.

### Data Analysis

Analyses were performed using MATLAB codes written by the authors. Methods for spectral and wavelet analysis were described previously (Igarashi et al., 2014).

#### LFP Analysis

##### Time-frequency analysis of power

The time-resolved power across frequencies was computed using a wavelet transform method. Signals were convolved by a family of complex Morlet’s wavelets *w(t, f)*, one for each frequency, as a function of time:

(1) $$w(t,\;f)=A\exp(-t^{2}/2\sigma_{t}^{2})\;\exp(2i\pi ft)$$

With $\sigma_{f}=\frac{1}{2}\pi \sigma_{t}$. The coefficient *A* was set at:

(2) $${\left(\sigma_{t}\sqrt{\pi }\right)}^{-1/2}$$

in order to normalize the wavelets such that their total energy was equal to 1. The family of wavelets was characterized by a constant ratio *f/σ_f_*, which was set to 7.

##### Normalization of LFP power

**Figure 1:**
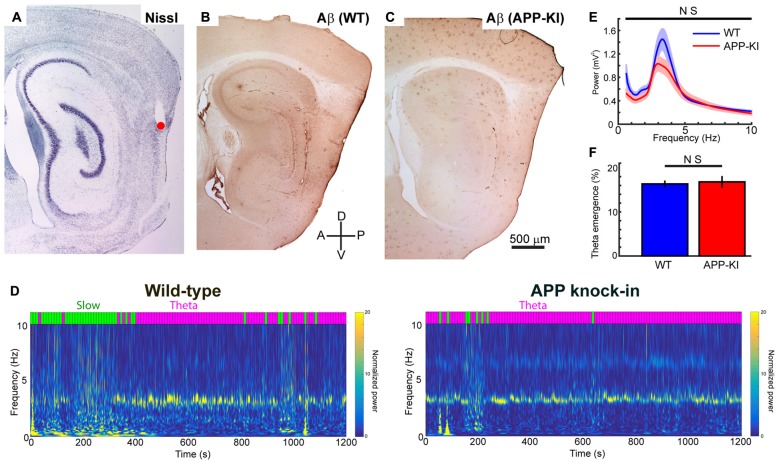
Amyloid precursor protein knock-in (APP-KI) mice showed normal theta state comparable to wild-type (WT) mice. **(A–C)** Representative sagittal brain sections showing the recording positon (red point) in medial entorhinal cortex (MEC; **A**) and Aβ immunostaining in WT mouse (**B**, WT) and APP-KI mouse (**C**, APP-KI). A, anterior; P, posterior, D; dorsal; V, ventral. Scale bar, 500 μm. **(D)** Time-resolved power spectrum in 0–10 Hz band from a representative WT mouse (left) and an APP-KI mouse (right). Power normalized by 5–10 Hz mean power is color-coded. Each 10-s time bin was divided into theta state (Theta) and slow wave state (Slow) and color-coded with magenta and green, respectively, shown on top of the spectra (see “Materials and Methods” Section). **(E)** Mean raw power spectra ± SEM (shading) in 0.4–10 Hz band during theta period in WT mice (*n* = 33 recording sessions) and APP-KI mice (*n* = 31 recording sessions). Comparison at each 0.2 Hz step showed no significant difference (*q* < 0.05, false discovery rate (FDR) corrected). **(F)** Emergence rate of theta period in WT mice (*n* = 33 recording sessions) and APP-KI mice (*n* = 31 recording sessions). There was no significant difference (*p* > 0.05, Mann-Whitney *U* test).

To control for impedance differences between tetrodes, LFP power was normalized, for each tetrode, to power in 5–10 Hz band, except for the analysis in Figure 1E.

##### Separation of LFP states

**Figure 2:**
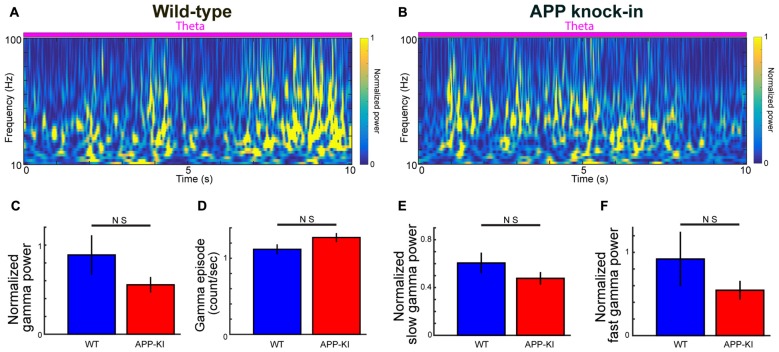
Gamma oscillations in APP-KI mice. **(A,B)** Time-resolved power spectrum in 10–100 Hz band during theta period from a representative WT mouse (left) and an APP-KI mouse (right). Power normalized by 5–10 Hz mean power is color-coded. **(C)** Normalized gamma power in 30–100 Hz band did not differ between WT mice (*n* = 33 recording sessions) and APP-KI mice (*n* = 31 recording sessions; *p* > 0.05, Mann-Whitney *U* test). **(D)** Emergence rate of gamma episode did not differ between WT mice (*n* = 33 recording sessions) and APP-KI mice (*n* = 31 recording sessions; *p* > 0.05, Mann-Whitney *U* test). **(E,F)** Normalized power of **(E)** slow gamma oscillation (30–50 Hz) and **(F)** fast gamma oscillations (55–100 Hz) did not differ between WT mice (*n* = 33 recording sessions) and APP-KI mice (*n* = 31 recording sessions; *p* > 0.05, Mann-Whitney *U* test).

**Figure 3:**
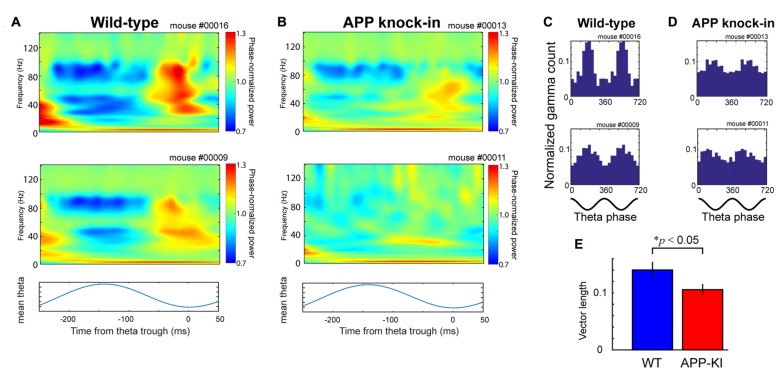
APP-KI mice showed impaired theta-gamma cross-frequency coupling. **(A)** Top and middle, time-resolved power spectra triggered at theta trough (*t* = 0) and averaged across all theta cycles from two representative WT mice. Bottom, averaged theta cycle. **(B)** Same as in **(A)**, but from two representative APP-KI mice. **(C,D)** Theta phase distributions of gamma oscillation maxima from **(C)** two representative WT mice and **(D)** two representative APP-KI mice. Count of gamma maxima is shown as normalized number for two gamma cycles (bottom; 0°, theta peak). **(E)** Mean vector length of theta phase distributions of gamma oscillation maxima for WT mice (*n* = 33 recording sessions) and APP-KI mice (*n* = 31 recording sessions). APP-KI mice showed reduced degree of cross-frequency coupling (*p* < 0.05, Mann-Whitney *U* test).

**Figure 4:**
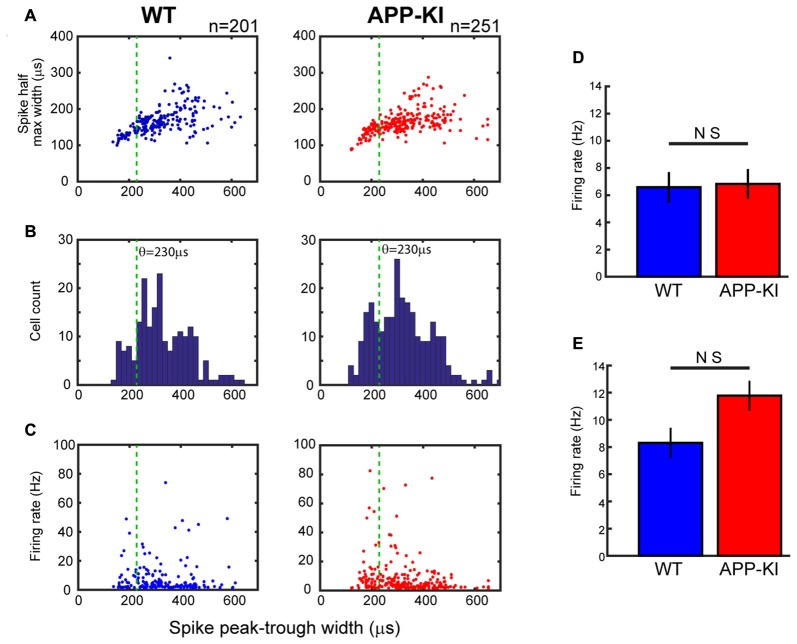
Neurons in APP-KI mice showed normal spike waveform and firing rate. **(A)** Plot of individual neurons for spike half-maximum width as a function of spike peak-trough width in WT mice (left) and APP-KI mice (right; *n* = 201 and 251 neurons, respectively). Green lines indicate 230 μs threshold used for separating interneurons and pyramidal neurons (see text). **(B)** Distributions of spike peak-trough width from WT mice (left) and APP-KI mice (right). Note that both distributions have a gap at 210–250 μs, which presumably represents the gap of different spike width between interneurons and pyramidal neurons. **(C)** Plot of individual neurons for mean firing rate during theta periods as a function of spike peak-trough width in WT mice (left) and APP-KI mice (right). **(D,E)** Mean firing rate for putative pyramidal neurons (**D**, *n* = 171 and 191 for WT and APP-KI) and interneurons (**E**, *n* = 30 and 60 for WT and APP-KI). No difference in firing rate was observed (Mann-Whitney *U* test).

**Figure 5:**
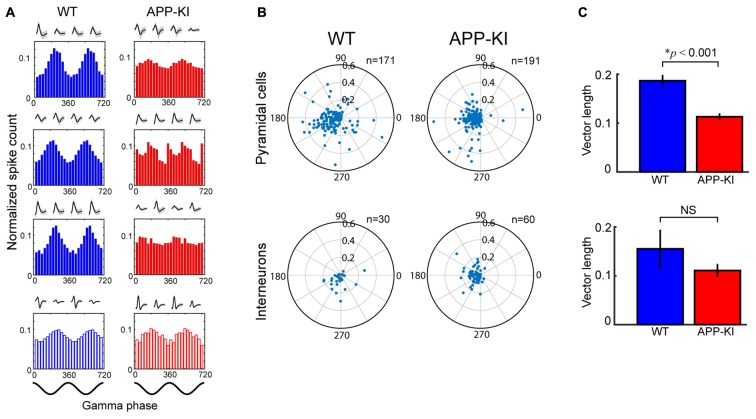
Pyramidal neurons in APP-KI mice showed impaired gamma phase locking. **(A)** Spike-time distributions of neurons across phase of gamma oscillation. Distributions of three representative pyramidal cells (Top) and an interneuron (Bottom) is shown for two gamma cycles with their waveforms recorded in each tetrode channel. Pyramidal cells in WT mice (left) showed larger peaks of phase distribution than those in APP-KI mice (right). Schematics of two cycles of gamma waveform are shown in the bottom (0°, gamma peak). **(B)** Polar plot of resultant vector angle and length computed from gamma phase distribution of individual pyramidal cells (top) and interneurons (bottom) in WT mice (left) and APP-KI mice (right). **(C)** Mean length of resultant vector in **(B)**. Pyramidal cells in APP-KI mice showed impaired degree of phase locking to gamma oscillations (*p* < 0.001, Mann-Whitney *U* test). Interneurons did not show significant difference (*p* > 0.05, Mann-Whitney *U* test).

**Figure 6:**
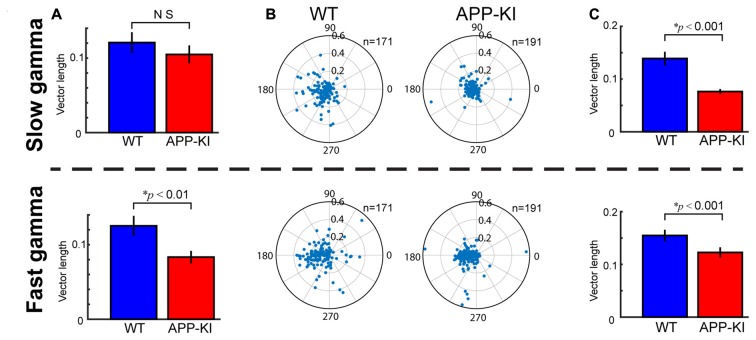
Fast gamma oscillations were more affected than slow gamma oscillations. **(A)** Degree of theta-gamma cross-frequency coupling, same as shown in Figure 3E, but for slow gamma oscillations (top) and fast gamma oscillations (bottom). Mean vector length of gamma maxima distribution was shown. APP-KI mice showed impaired cross frequency coupling between theta and fast gamma oscillations (*p* < 0.01), but not for slow gamma oscillations (*p* > 0.05, Mann-Whitney *U* test). **(B)** Polar plot of phase distribution for individual pyramidal cells, same as shown in Figure 5B, but for slow gamma phase (top) and fast gamma phase (bottom). **(C)** Mean vector length of phase locking for pyramidal neurons, same as shown in Figure 5C, but for slow gamma (top) and fast gamma (bottom). Pyramidal neurons in APP-KI mice showed impaired phase locking to both slow and fast gamma oscillations (*p* < 0.001, Mann-Whitney *U* test).

**Figure 7:**
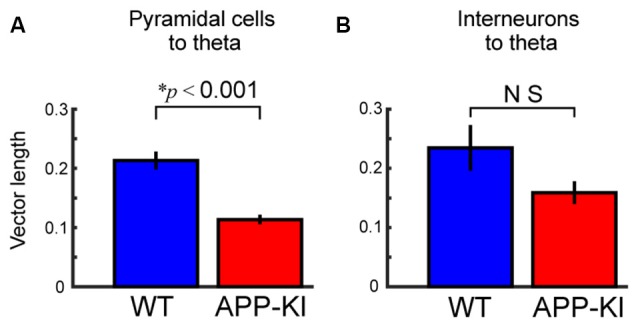
Pyramidal neurons in APP-KI mice showed impaired theta phase locking. **(A,B)** Mean vector length of phase locking for pyramidal neurons **(A)** and interneurons **(B)**, same as shown in Figure 5C, but for theta oscillations. Pyramidal neurons in APP-KI mice showed impaired phase locking to theta oscillations (*p* < 0.001), but interneurons did not (*p* > 0.05, Mann-Whitney *U* test).

We divided LFP data into 10 s bins. For each bin, ratio of the power of theta oscillation (2.5–4.5 Hz) to the power of slow wave oscillation (0.5–1.5 Hz) was calculated. We defined bins with a ratio ≥1 as theta states and a ratio <1 as slow wave states. We used data recorded only during theta states in the analyses in Figures 2–7.

##### Bandpass filtering

An acausal (zero phase shift), frequency domain, finite impulse response bandpass filter was applied to the signals. For theta filtering, 1.5 and 2.5 Hz were chosen for the stopband and passband, respectively, for the low cut-off frequencies; 4.5 and 5.5 Hz were chosen for passband and stopband high cut-off frequencies. For filtering of gamma oscillations, 28 and 30 Hz were chosen for the stopband and passband, respectively, for the low cut-off frequencies; 100 and 102 Hz were chosen for passband and stopband high cut-off frequencies. For filtering of slow gamma oscillations, 28 and 30 Hz were chosen for the stopband and passband, respectively, for the low cut-off frequencies; 50 and 52 Hz were chosen for passband and stopband high cut-off frequencies. For filtering of fast gamma oscillations, 53 and 55 Hz were chosen for the stopband and passband, respectively, for the low cut-off frequencies; 100 and 102 Hz were chosen for passband and stopband high cut-off frequencies.

##### Selection of theta cycles

Theta cycles were selected by bandpass filtering the signal from 2.5–4.5 Hz and selecting local minima in the filtered signal (that is, *θ*(*t* − 1) > *θ*(*t*) and *θ*(*t* + 1) > *θ*(*t*)). Segments of the recording were collected and defined as a theta cycle if the time between detected points fell within a range criterion that corresponded to the period of a ~3.5-Hz theta cycle. Local minima of detected theta cycles were required to be separated by at least 200 ms.

##### Detection of oscillation episodes

To extract periods of gamma oscillatory activity in the LFP, we first computed time-varying power within the frequency bands for each recording. Power at each time point was averaged across the frequency range to obtain time-varying estimates of oscillatory power. Time points were collected when the power exceeded 2 SD of the time-averaged power. Time windows, 160 ms in length, were cut around the identified time points. In each 160-ms segment, the maxima of gamma oscillatory amplitude were determined from the gamma bandpass filtered versions of the recordings. Duplicated gamma oscillatory periods, a common consequence of extracting overlapping time windows, were avoided by discarding identical maxima values within a given gamma oscillatory subtype and further requiring that maxima of a given subtype be separated by at least 100 ms. Individual gamma oscillatory windows were finally constructed from the original, non-bandpass filtered recordings as 400 ms long windows centered around the gamma oscillatory amplitude maxima.

##### Relationship of gamma to theta phase

LFP recordings were bandpass-filtered in the theta range (2.5–4.5 Hz), and theta phases for each time point were estimated using the Hilbert transform function from the Signal Processing Toolbox in MATLAB. Theta phases at the time points associated with gamma maxima (determined as described above) as well as theta phases for spikes were collected. Theta phases for each gamma oscillatory event were sorted into 30° bins, allowing the phase distribution of each event to be determined. For a given recording, the distributions of gamma oscillations were normalized by dividing the bins by the total number of gamma oscillatory episodes within a given recording. In this analysis, and in all analyses involving oscillation phase, the oscillation peak was defined as 0°.

##### Time–frequency representation of power across individual theta cycles

Time-varying power in 2-Hz-wide frequency bands, from 2 Hz to 140 Hz, was obtained for individual theta cycles using the wavelet transform method described above. Time frequency representations for multiple theta cycles recorded from the same site and session within the same animal were then averaged.

##### Strength of theta-gamma coupling

The theta phase at the time of gamma oscillation maxima occurrence was calculated by bandpass-filtering the LFP in the theta range, performing a Hilbert transform on the filtered signal, and then locating theta phase of individual gamma oscillation maxima. Resultant vectors were calculated from the phase distributions gamma maxima. Lengths of resultant vectors were used as an index for the strength of theta-gamma coupling.

#### Spike Analysis

##### Spike sorting and cell classification

Spike sorting was performed offline using graphical cluster-cutting software described previously (Igarashi et al., 2014). Putative excitatory cells were distinguished from putative interneurons by using spike width at half-maximum amplitude and spike peak-trough width (Barthó et al., 2004).

##### Phase-locking of spikes to gamma oscillations and theta oscillations

The oscillatory phase at the time of spike occurrence was estimated. This was performed by first bandpass-filtering the LFP, performing a Hilbert transform on the filtered signal, and then extracting the phase component at the spike times. Cells were considered to be phase-locked if their phase distribution differed significantly from a uniform distribution (*P* < 0.05, Rayleigh test). Resultant vectors were calculated from the distributions of phase component. Lengths of resultant vectors were used as an index for the strength of phase-locking.

### Statistics

Mean ± SEM are provided for all analyses. All statistical testing assumed a non-parametric distribution and Mann-Whitney *U* test was used. Power analysis was performed using Matlab with power set at 0.9.

### Histology

After completion of the recordings, microdrives and electrodes were fixed to the skull using dental cement. Mice received an overdose of urethane, and were perfused intracardially with saline and 4% formaldehyde. The brains were then removed and stored in formaldehyde. For verification of tetrode locations, the brains were frozen, cut at 40 μm thickness on a cryostat, and stained with cresyl violet. Sections adjacent to the tetrode position were immunostained with anti-Aβ monoclonal antibody (6E10, 1:1000; Covance) and visualized using avidin–biotin–peroxidase complex (ABC) histochemical protocol (ABC Elite; Vector Laboratories). Digital photomicrographs were acquired with a Zeiss Axioplan microscope equipped with a digital camera.

## Results

### APP Knock-in Mice Showed Normal Theta State Comparable to Wild-Type Mice

Consistent with previous reports describing Aβ deposition at 4-months old (Saito et al., 2014), we found prominent deposition of Aβ in the dorsal part of MEC of 5-month old APP-KI mice (Figure 1). Age-matched WT mice did not show strong Aβ labeling in MEC. We recorded spike and LFP activity from dorsal part of MEC, where previous recording studies showed gamma oscillations (Chrobak and Buzsáki, 1998; Colgin et al., 2009). During recording, we progressively advanced recording tetrodes through layers III and II of MEC, and performed 64 recording sessions from a total of 10 mice (*n* = 5 WT mice and *n* = 5 APP-KI mice). Each session contained a distinct set of recorded neurons. We recorded 452 layer II/III MEC neurons in total (*n* = 201 and 251 neurons from WT and APP-KI, respectively).

Previous recording studies from freely behaving animals showed that running speed of animals alters the power and frequency of gamma oscillations in CA1 (Chen et al., 2011; Ahmed and Mehta, 2012) and MEC (Igarashi et al., 2014). To eliminate the effect of animal movement and assess gamma oscillations under stable conditions, we performed recordings from mice lightly anesthetized with urethane. Urethane anesthesia has long been used as a preferred anesthetic to study various aspect of entorhinal-hippocampal functions including theta and gamma oscillations (Buzsáki and Eidelberg, 1983; Bland, 1986; Quilichini et al., 2010). During urethane anesthesia, animals show two spontaneously alternating states: the “theta state” where animals exhibit theta oscillations (3–7 Hz), and the “slow wave state” where 0.5–1.5 Hz slow oscillations are prominent (Klausberger et al., 2003; Murakami et al., 2005; Quilichini et al., 2010). Although the frequency of theta oscillations in anesthetized animals is relatively slower than the 6–12 Hz theta oscillations in awake animals, animals in the anesthetized theta state show MEC gamma oscillations that share most of the properties of those in awake behaving animals (Chrobak and Buzsáki, 1998; Quilichini et al., 2010). Consistent with previous studies, our recordings from WT mice showed alternating states with 2.5–4.5 Hz theta oscillations and 0.5–1.5 Hz slow wave oscillations (Figure 1D). We calculated the ratio of theta power to slow wave power for each 10 s bin, and classified the bins into theta state (Power_theta_/Power_slow_ ≥ 1) and slow wave state (Power_theta_/Power_slow_ < 1; Figure 1D, see “Materials and Methods” Section). APP-KI mice also showed the two alternating states, with a theta oscillation range similar to that of WT mice (Figures 1D,E). We then compared the raw power of oscillations in the 0.4–10 Hz frequency range at every 0.2 Hz step. Although there’s a slight shift in the power of theta oscillations between WT and APP-KI mice, no significant difference was observed in the power of oscillations in the 0.4–10 Hz frequency range (Figure 1E; *q* > 0.05, false discovery rate (FDR) corrected for multiple comparison; *n* = 31 WT and *n* = 33 APP-KI recording sessions). Emergence rate of theta states did not differ between WT and APP-KI mice (Figure 1F; WT: 16.3 ± 0.8%; APP-KI: 16.8 ± 1.3% of time in recording sessions; *p* > 0.05, Mann–Whitney *U* test; *n* = 31 WT and *n* = 33 APP-KI recording sessions). These data indicate that APP-KI mice have theta states that are comparable to those of WT mice.

### Theta-Gamma Cross-Frequency Coupling Was Impaired in APP Knock-in Mice

We next analyzed gamma oscillations. In the following analyses, we focused on only gamma oscillations in theta states. Several previous studies on gamma oscillations in AD mouse models compared raw power of gamma oscillations. However, raw power of gamma oscillations is generally not informative, because gamma power is normally smaller than theta power, while the variance of electrode impedance and extracellular microenvironments around the electrode tip can significantly affect recorded gamma power. Normalization using internal reference typically offers more reliable controlling of gamma power (Igarashi et al., 2014; Iaccarino et al., 2016). Taking advantage of the fact that 5–10 Hz power was relatively stable during the theta state and invariant across recordings (Figure 1E; *q* > 0.05, FDR corrected for multiple comparison; *n* = 31 WT and *n* = 33 APP-KI recording sessions), we calculated normalized gamma power: raw power divided by 5–10 Hz mean power in the same session (see “Materials and Methods” Section). Figures 2A,B show examples of transient and repetitive gamma oscillations from WT and APP-KI mice, respectively. Gamma oscillations in ~30–100 Hz range were prominent during theta states in both strains. We then compared the normalized power of 30–100 Hz gamma oscillations between WT and APP-KI mice. Although APP-KI mice showed mildly reduced gamma power as compared with WT mice, the difference was not significant (Figure 2C; WT: 0.89 ± 0.23; APP-KI: 0.56 ± 0.09; *p* > 0.05, Mann-Whitney test; *n* = 31 WT and *n* = 33 APP-KI recording sessions). Power analysis showed that we have enough sample size to detect significance expected from the difference between WT and APP-KI mouse (*n* ≥ 28), suggesting that the mild difference observed in our data set resulted from the variability of gamma power. The emergence rate of gamma oscillation episodes was comparable between WT and APP-KI mice (Figure 2D; WT: 1.12 ± 0.07; APP-KI: 1.27 ± 0.06; *p* > 0.05, Mann-Whitney test; *n* = 31 WT and *n* = 33 APP-KI recording sessions). The power of gamma oscillations did not differ even they were separately compared in slow gamma band (30–50 Hz) and fast gamma band (55–100 Hz; Figures 2E,F; slow gamma, WT: 0.61 ± 0.09; APP-KI: 0.48 ± 0.05; *p* > 0.05, Mann-Whitney test; fast gamma, WT: 0.92 ± 0.33; APP-KI: 0.55 ± 0.11; *p* > 0.05, Mann-Whitney test; *n* = 31 WT and *n* = 33 APP-KI recording sessions).

Next, we evaluated if the temporal structure of gamma oscillations is affected in APP-KI mice by analyzing theta-gamma cross-frequency coupling. We first cropped individual theta cycles and calculated a mean of oscillatory activities (Figures 3A,B). Our results showed that the peak of 30–100 Hz gamma oscillations, observed at the falling phase to trough of theta oscillations in WT mice, was diminished in APP-KI mice (Figure 3B). The theta phase histogram of gamma episode count indicated that APP-KI mice had gamma oscillations that were more scattered across theta phases than those in WT mice (Figures 3C,D). This notion was confirmed by the decreased vector length of phase distribution in APP-KI mice (Figure 3E; WT: 0.142 ± 0.010; APP-KI: 0.103 ± 0.008; *p* < 0.05, Mann-Whitney test; *n* = 31 WT and *n* = 33 APP-KI recording sessions). These data indicate that theta-gamma cross-frequency coupling was impaired in MEC of APP-KI mice. This suggests the temporal structure of gamma oscillations was impaired in APP-KI mice, although the power and the number of gamma oscillations were not significantly affected.

The LFP analyses above were performed using 64 LFP recording sessions (*n* = 31 WT and *n* = 33 APP-KI recording sessions). These sessions were obtained at different recording sites that were apart at least 80 μm with no overlapping spikes. Because LFP signals are generally a combination of current from oscillating dipoles and volume conduction (Kajikawa and Schroeder, 2011), we further asked if our conclusions obtained above are valid in an assumption that all LFP data from the same animal were dominated with volume conducted currents. We averaged all data taken from a single animal to make one data point, and performed statistics using only one sample from each animal (*n* = 5 WT and *n* = 5 APP-KI recording data). No significant difference was observed in the power of oscillations in the 0.4–10 Hz frequency range (*q* > 0.05, FDR corrected for multiple comparison). Emergence rate of theta states did not differ between WT and APP-KI mice (WT: 15.9 ± 0.9%; APP-KI: 17.2 ± 0.9% of time in recording sessions; *p* > 0.05, Mann–Whitney *U* test). No significant difference was observed in the power of oscillations in the 5–10 Hz frequency range (*q* > 0.05, FDR corrected for multiple comparison). No significant difference was observed in the power of oscillations in the 30–100 Hz frequency range (WT: 0.80 ± 0.43; APP-KI: 0.59 ± 0.19; *p* > 0.05, Mann-Whitney test). Significant decrease of vector length of phase distribution in APP-KI mice still existed (WT: 0.151 ± 0.012; APP-KI: 0.082 ± 0.010; *p* < 0.05, Mann-Whitney test). Thus, all conclusions obtained using different recording positions as independent measure were valid in these analyses using averaged LFP data.

### Gamma Phase Locking of Spikes of Pyramidal Cells Was Impaired in APP Knock-in Mice

We next asked if the spike activities of MEC layer II/III neurons were affected in APP-KI mice. We first checked spike waveforms using two parameters previously used for separating putative pyramidal cells and interneurons (Barthó et al., 2004; Mizuseki et al., 2009). Figures 4A,B show the plot of half-maximum width as a function of peak-trough width. APP-KI mice and WT mice showed a similar distribution of peak-trough width (*p* > 0.05 for the both parameters, Kolmogolov-Smirnov test; *n* = 201 neurons from WT and *n* = 251 neurons from APP-KI). Because the distribution of peak-trough width showed a gap at ~230 μs in both WT and APP-KI mice (Figure 4B), we set 230 μs as a threshold for separating pyramidal cells from interneurons. Recorded samples were classified into 171 pyramidal cells and 30 interneurons in WT mice, and 191 pyramidal cells and 60 interneurons in APP-KI mice. We then compared firing rate of spontaneous spike activity. The mean firing rate of both pyramidal cells and interneurons during theta state did not differ between the two strains (Figures 4C–E; pyramidal cells, WT: 6.5 ± 1.1; APP-KI: 6.8 ± 1.1; interneurons, WT: 8.3 ± 2.3; APP-KI: 11.8 ± 2.6; *p* > 0.05 for both cell types, Mann-Whitney test; *n* = 171 and *n* = 191 pyramidal neurons and *n* = 30 and *n* = 60 interneurons from WT and APP-KI, respectively).

We then evaluated if the gamma phase locking of spiking activity was affected in APP-KI mice. Figure 5A shows a gamma phase histogram of spikes of representative neurons in WT and APP-KI mice. While spiking activity of pyramidal neurons preferentially occurred at the gamma trough in WT mice, APP-KI mice showed spiking activity scattered across gamma phases. Reduced phase locking of pyramidal cells in APP-KI mice was also evident in the polar plot of phase-vector length for individual neurons (Figure 5B). A comparison of mean vector lengths showed that MEC layer II/III pyramidal cells in APP-KI mice showed impaired gamma phase locking (Figure 5C; WT: 0.187 ± 0.012; APP-KI: 0.113 ± 0.007; *p* < 0.001, Mann-Whitney test; *n* = 171 and *n* = 191 cells from WT and APP-KI). Interneurons did not show a significant reduction of phase locking (Figure 5C, WT: 0.155 ± 0.039; APP-KI: 0.111 ± 0.014; *p* > 0.05, Mann-Whitney test; *n* = 30 and *n* = 60 cells from WT and APP-KI), but power analysis showed that our sample size was fewer than that needed to detect significance (*n* ≥ 245), leaving a possibility that interneurons also have reduction in gamma phase locking. These data indicate that the entrainment of spike activity by gamma oscillations was impaired for layer II/III pyramidal neurons in APP-KI mice.

### Fast Gamma Was More Affected than Slow Gamma

Fast gamma oscillations are prominent in the dorsal part of MEC of freely moving rats (Colgin et al., 2009). We assessed if the temporal structure of gamma oscillations were similarly impaired for slow gamma (30–50 Hz) and fast gamma (55–100 Hz) oscillations, by repeating the same analyses for slow and fast gammas separately. We found that, while theta-fast gamma coupling was affected in APP-KI mice, theta-slow gamma cross-frequency coupling was not significantly altered (Figure 6A; slow gamma, WT: 0.121 ± 0.014; APP-KI: 0.105 ± 0.012, *p* > 0.05; fast gamma, WT: 0.125 ± 0.013; APP-KI: 0.083 ± 0.008, *p* < 0.01; Mann-Whitney test; *n* = 31 for WT and *n* = 33 for APP-KI recording sessions). Spike phase locking of pyramidal neurons to slow gamma and fast gamma were both impaired in APP-KI mice (Figures 6B,C; slow gamma, WT: 0.139 ± 0.013; APP-KI: 0.076 ± 0.005, *p* < 0.001; fast gamma, WT: 0.155 ± 0.011; APP-KI: 0.123 ± 0.010, *p* < 0.001; Mann-Whitney test; *n* = 171 and *n* = 191 cells from WT and APP-KI). These data suggest that the temporal entrainment by fast gamma oscillations was more severely impaired than by slow gamma oscillations.

### Theta Entrainment of Spikes Was also Affected in Layer II/III Pyramidal Cells

Our results indicate the temporal organization between theta and gamma oscillations, and temporal structure between spikes of layer II/III pyramidal cells and gamma oscillation were both disrupted in APP-KI mice. To clarify if the temporal organization between spikes of pyramidal cells and theta oscillations is also affected, we assessed phase locking of spikes to theta oscillations. The result showed that the spikes of layer II/III pyramidal cells exhibited significant reduction in theta phase locking (Figure 7A; WT: 0.213 ± 0.015; APP-KI: 0.114 ± 0.008; *p* < 0.001, Mann-Whitney test; *n* = 171 and *n* = 191 cells from WT and APP-KI). Interneurons did not show a significant reduction of theta phase locking (Figure 7B; WT: 0.235 ± 0.039; APP-KI: 0.159 ± 0.019; *p* > 0.05, Mann-Whitney test; *n* = 30 and *n* = 60 cells from WT and APP-KI), although a power analysis showed that our sample size was fewer than that needed to detect significance (*n* ≥ 85), thus leaving a possibility that interneurons also have reduction in theta phase locking. These data suggest that not only gamma oscillations, but also the spike timing of pyramidal cells lose the phase-locking organization to theta oscillations.

### Impairment of Theta-Gamma Coupling Was Prominent in Layer III

We showed the impairment of theta-gamma coupling and phase locking by analyzing layers II and III altogether. However, since it was reported that MEC layers II and III have different magnitude of cross-frequency coupling, gamma phase locking and theta phase locking (Mizuseki et al., 2009; Quilichini et al., 2010), the impairment we observed might result from sampling bias between layers II and III. We thus analyzed layers II and III separately (Supplementary Figure S1). Layer positions were estimated by the traveling distance of tetrodes during recording and the thickness of layers in stained sections. The result showed that the impairment of theta-gamma coupling was again observed in layer III (Supplementary Figure S1A; WT: 0.161 ± 0.024; APP-KI: 0.108 ± 0.014, *p* < 0.05; Mann-Whitney test, *n* = 18 WT and *n* = 21 APP-KI recording sessions). Although we did not see significant impairment in layer II (WT: 0.121 ± 0.017; APP-KI: 0.104 ± 0.014, *p* > 0.05, *n* = 13 WT and *n* = 12 APP-KI recording sessions), this could be due to the smaller sample size resulting from the subdivision into layer II and III, because power analysis indicates that samples with more than *n* ≥ 143 is needed to detect the significance with the difference observed here. Alternatively, this may reflect the fact that theta-gamma coupling is strongest in layer III (Quilichini et al., 2010), and the reduction was detectable only in this layer. On the other hand, the impairment of gamma and theta phase locking of pyramidal spikes were observed in both layers II and III even if they were separately analyzed (Supplementary Figures S1B,C; LII pyramids to gamma, WT: 0.170 ± 0.011; APP-KI: 0.111 ± 0.011; LIII pyramids to gamma, WT: 0.203 ± 0.021; APP-KI: 0.114 ± 0.009; LII pyramids to theta, WT: 0.240 ± 0.027; APP-KI: 0.094 ± 0.006; LIII pyramids to theta, WT: 0.185 ± 0.013; APP-KI: 0.145 ± 0.019; *p* < 0.001 for all comparisons, Mann-Whitney test; *n* = 84 WT and *n* = 67 APP-KI Layer II cells; *n* = 87 WT and *n* = 124 APP-KI Layer III cells).

## Discussion

In this study, we showed that theta-gamma cross-frequency coupling and gamma phase locking of MEC layer II/III pyramidal cells were impaired in APP-KI mice, as compared with age-matched WT mice. Our results indicate that, even though the power of the gamma oscillations or the emergence rate of gamma episodes were unaffected, temporal organization of gamma oscillations was significantly disrupted in APP-KI mice. While gamma oscillations occurred at specific phases of theta oscillations in healthy subjects, gamma oscillations in the AD mouse model occurred randomly across various theta phases. Spiking activity also occurred in a scattered manner across both theta and gamma phases.

Computational studies suggest the roles of gamma oscillations in grouping of neuronal spike activities into assembly and in information transfer across discrete brain regions (Lisman and Idiart, 1995; Lisman, 2005). Recording studies from subjects engaging in memory tasks showed that theta-gamma cross-frequency coupling in hippocampus and EC correlated well with subjects’ task performance, suggesting a role of theta-gamma cross-frequency coupling in the enhancement of memory encoding (Axmacher et al., 2010; Igarashi et al., 2014). Impaired temporal structure in the MEC of APP-KI mice may cause reduction of information transfer in the entorhinal-hippocampal circuit and result in the impairment of spatial memory performance previously observed in APP-KI mice (Saito et al., 2014).

What are the cellular mechanisms that cause reduced temporal organization of gamma oscillations in APP-KI mice? In layer II of MEC, excitatory stellate cells are interconnected mainly via inhibitory interneurons (Couey et al., 2013). This type of excitatory-inhibitory loop is suggested to serve as a generator of the gamma oscillations (Wilson and Cowan, 1972; Cunningham et al., 2003). Indeed, a recent patch-clamp study using MEC slices showed that optogenetic excitation of MEC layer II neurons at theta frequency pattern generated membrane potential oscillations at gamma frequency (Pastoll et al., 2013). The summation of these activities led to generation of field potential gamma oscillations occurring at the trough of theta oscillations. Thus, we speculate that APP-KI mice have a dysfunction in the MEC excitatory-inhibitory feedback loop, and such dysfunction impairs theta-structured gamma oscillations and spiking activities. Evidence from a transgenic mouse model of AD showed reduced gamma in parietal cortex was caused by decreased sodium channel function in palvalbumin-expressing interneurons (Verret et al., 2012). However, the role of parvalbumin-expressing interneurons in gamma reduction is still unclear in MEC of APP-KI model. Further studies will be needed to point out the molecular and cellular mechanisms for gamma impairment.

In conclusion, our findings provided the first evidence of impaired *in vivo* gamma oscillations in the EC of AD mouse model. Our results point to gamma oscillations as a possible mechanism for cognitive dysfunction in the entorhinal-hippocampal network. Future studies using behaving mice as well as *in vivo* recording from other half of EC, the lateral EC, will clarify the role of gamma oscillations in AD.

## Author Contributions

KMI designed experiments and analyses; TN, TNL, AYP and KMI performed the *in vivo* experiments; TN and KMI performed the analyses; MK and KMI performed the histology; TS and TCS provided APP-KI mice; TN and KMI wrote the article with input from all authors.

## Conflict of Interest Statement

The authors declare that the research was conducted in the absence of any commercial or financial relationships that could be construed as a potential conflict of interest.
