# Supplementary figures and images for: Impaired In Vivo Gamma Oscillations in the Medial Entorhinal Cortex of Knock-in Alzheimer Model
Source: Front Syst Neurosci. 2017 Jun 30;11:48. doi: 10.3389/fnsys.2017.00048 (PMC5491963; doi:10.3389/fnsys.2017.00048)

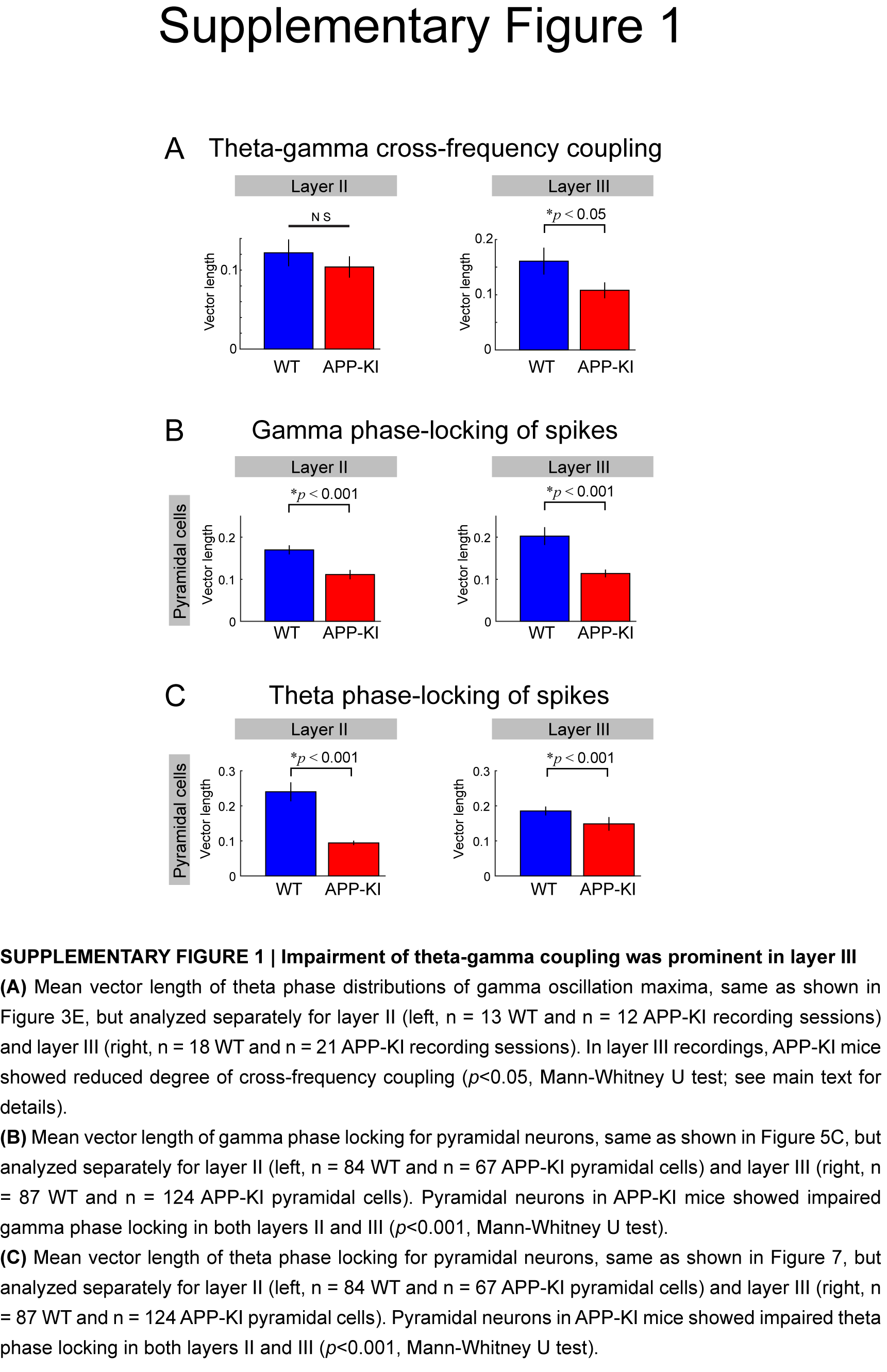

Supplement: Supplementary file 1 [file Image_1.tif]
